# Case of Extra-Uterine Pregnancy

**Published:** 1850-09

**Authors:** Carter P. Johnson

**Affiliations:** Professor of Anatomy and Physiology in the Medical Department of Hampden Sidney College, Richmond, Va.


*Case of Extra-Uterine Pregnancy.* Reported by Carter P. Johnson, M. D., Professor of Anatomy and Physiology in the Medical Department of Hampden Sidney College, Richmond, Va.

The patient was a negro woman about 30 years of age, admitted into the Infirmary of the Medical College, from Souria County, Va., on May 22d. The following history of her case, up to the date of her admission, has been kindly furnished me, since the operation, by Dr. William Beadles, her attendant Physician:

She had enjoyed good health until the commencement of the present affection. She has been married for several years, but has borne no children, though her catamenia had always made their appearance regularly. She menstruated for the last time about the 1st of December, 1848. On the 18th of December, she was taken with violent pain in the lower portion of the abdomen, recurring in paroxysms and accompanied by nausea and vomiting. The affection was referred by the physician in attendance "to the uterus or its appendages, and was quite relieved in the course of three or four days by pursuing a moderately antiphlogistic and sedative treatment." For several months after this period, she had repeated attacks of a similar character, and during the whole of the ensuing year, she continued very unwell and poorly. In the mean time a tumor made its appearance in the hypogastric region, and went on gradually to increase in the same manner as the uterine tumor in ordinary cases of pregnancy. Her breasts also enlarged, and towards, the latter period of her supposed pregnancy, secreted a certain amount of milky fluid.

During the month of August, 1849, about the time she expected her confinement, she experienced a sanguineous discharge from the vagina, accompanied by considerable pain. Thinking herself in labor, she summoned a midwife, and made her preparations in anticipation of the immediate occurrence of the expected event. The pain, however, at one time severe, gradually subsided and disappeared entirely in the course of three or four days. The discharge, which was attended with the escape of coagula, continued about a week. About three weeks after this, she was again seen by Dr. Beadles, in connection with Dr. Fox, and "was found confined to her bed and helpless, mainly on account of having *phlegmasia dolens*," of which she was relieved in the course of a few weeks. From that time to the date of her admission, she has been occasionally annoyed by pains in the loins and in the region of the tumor. Within the last few months, these have very greatly diminished, and since the beginning of the present year, her general health has improved sufficiently to allow her to walk about the neighborhood at short distances, not exceeding a mile. She has frequently felt what she conceived to be the movements of a child, and she states that she has *continued to feel them until within six weeks past.*

At the date of her admission, the case presented the following symptoms. Her general appearance was healthy, her skin soft, pulse natural, secretions generally normally performed. The abdomen presents a large, globose tumor, remarkably uniform, looking very much like a huge mammary gland, the retracted nipple being represented by the umbilicus. The tumor is more prominent than the uterine tumor at the ninth month of utero-gestation, and occupies a higher position in the abdomen. When the patient is in the erect position, the integuments are rendered very tender, and the tumor is with difficulty circumscribed; in the recumbent posture, the integuments being somewhat relaxed, it may be moved slightly in all directions, and is discovered evidently not to fill up the entire peritoneal cavity. Upon succussion, fluctuation is discovered, though very obscure. The tumor presents *no irregularity* of surface, and impinges *equally* on both sides of the median line.

An examination *per vaginam* gave no indications of any disease of the uterus or of previous impregnation. A very obscure fluctuation was perceptible through the walls of the vagina just behind the posterior lip of the os uteri.

Upon consultation with my colleagues, Professors Gibson and Tucker, (to both of whom I am very much indebted for valuable assistance in the subsequent operation,) it was determined, as the case was very obscure, and neither of us was fully satisfied as to its true nature, to make an exploratory incision into the tumor, and to be guided in the subsequent steps of the operation by the information which such an incision would afford us.

Accordingly, on the 25th of *May*, at 11 o'clock, assisted by Professors Gibson and Tucker, and Dr. Peticolas, and in the presence of a number of medical students, I proceeded to the operation.

The patient being placed upon her back, an incision was made about one inch below the imbilicus in the linea alba, through the integuments and superficial fascia for the space of about fifteen lines. The tendons of the abdominal muscles were then carefully incised until they were completely divided; the finger was then passed in, and came in contact with what I took to be the omentum very much thickened, on breaking through which, an entrance was effected into a cavity, from which from twelve to sixteen ounces of sero-purulent matter was discharged. On examining the interior of the cavity with the finger, a solid body was felt on both sides of the median line, distinctly moveable; the incision however was not large enough to permit a satisfactory examination as to its character.

At this stage of the operation, a consultation was again held, and at the suggestion of Dr. Gibson, it was determined to prolong the incision so as to enable us to extract the contents of the cavity, if practicable. Before proceeding further, an attempt was made to place the patient under the influence of chloroform, but was desisted from at her earnest entreaty. I then carried the incision downwards to within an inch of the pubes, and upwards about two inches above the umbilicus, when upon opening the lips of the wound a large and well grown fœtus was discovered compactly stowed away in this extra-uterine cavity. The head of the child looked towards the left iliac fossa, the breach towards the right hypochondriac region, the back towards the abdomen of the mother; the thighs were flexed upon the abdomen, and the legs upon the thighs. With but little difficulty, the fœtus was separated from its singular resting place, and delivered. The cord, which was entirely flaccid and bloodless, attached itself to the inner portion of the sac along the lower part of the anterior parietes of the abdomen on the left side. The placenta was atrophied to a considerable extent, and was attached along almost the whole of the anterior and upper portion of the sac. Its removal constituted the next stage in the operation, and was accomplished by separating, first those portions along the edges of the incision, by insinuating the fingers between them and the sac to which they were attached, and then effecting its removal precisely as we would remove an adhering uterine placenta. The vessels passing between the placenta, and the walls of the containing sac were very numerous, but were, and apparently had been, for some time, completely impervious, presenting, as the placental mass was torn off, the appearance of small fibrous cords.

Having separated the placenta, the cavity which contained the fœtus and its appendages was found to be surrounded by a very dense sac of plastic lymph, intimately adherent to the parietes of the abdomen anteriorly, but superiorly, posteriously and inferiorly, completely separating the cavity and its contents from the abdominal and pelvic viscera.

During the operation, an artery, a lateral branch of the left epigastric, about the size of the epigastric under ordinary circumstances, was cut and required a ligature.

Having cleansed the cavity as thoroughly as possible, the edges of the wound were brought together by sutures and adhesive straps, a large bandage firmly applied, and the patient put to bed in a very comfortable condition, having borne the protracted operation with great fortitude. She was ordered seventy-five drops of laudanum in a little brandy and water.

The child was very fully developed and perfectly formed, except that both feet presented "talipes volgus," the left slightly, the right to a considerable extent. The posterior fontanel was completely closed, the anterior was nearly as much open as usual. Its weight, with the placenta, which weighed only a few ounces, was *nine pounds.* It had evidently been dead for some time, as was inferred from the state of the placental bloodvessels, from the cod, from the condition of the skin, the cuticle being separated in mrost parts of the body, and from the eyes, which were deeply sunken in the orbits. There was no fœtor.

At 7 o'clock P. M. patient was quiet, had not slept, but was comfortable—pulse 94; skin cool and moist.

*May* 26th. Passed a quiet night, sleeping during the latter part; skin moist but warm; tongue moist and clean; pulse 102, soft and compressible; abdomen, soft and not tender to the touch; severe soreness about the wound; at 9 P. M. sleeping quietly; respiration good.

27th. Passed a comfortable night, sleeping throughout; perspired freely; tongue moist and furred; pulse 120; respiration somewhat hurried; abdomen flaccid and entirely free from pain; wound looks well. At 12 o'clock had slight chilliness; 9 o'clock P. M., perspiring freely; pulse 140; respiration more hurried; severe uneasiness in head; no pain in the abdomen. Ordered Calomel grs. xv., Rhubarb grs. x. M. To be taken immediately.

*May* 28th. Has been quiet during the night, though she has slept but little; pulse 120; skin soft and warm; tongue cleaner and moist; abdomen flaccid and free from pain; wound looks well and is discharging slightly; medicine has not operated. Ordered enema of gruel and sweet oil. 9 o'clock P. M. Enema has brought away two copious and consistent passages, giving great relief to the patient; head easy; pulse 120; skin moist; respiration 30.

29th. Slept well after 12 o'clock. This morning had another consistent evacuation; feels very comfortable; pulse 112; tongue moist; skin cool, and less tendency to perspiration; slight giddiness of head; no pain of the abdomen; wound looks well and discharges moderately; in the evening pulse rose to 120.

30th. Slept well; countenance improving; pulse 106; tongue cleaner; wound discharging, but not abundantly; appetite improving; no uneasiness of head; bowels opened during the day.

31st. and *June* 1st. The ligature and all the sutures removed.

5th. Has continued to improve in every respect up to this time, with the exception of slight sore throat, which is now relieved. The wound is discharging freely healthy pus, the edges have a florid and healthy appearance; pulse 96; ordered infusion of hops, a wine-glass full three times a day.

8th. Appetite improving; wound looks well; at its upper portion the opposite edges have adhered; discharges a considerable quantity of healthy pus; pulse 98 and more feeble. Ordered Vallet's proto-carbonate of iron 5 grs. three times a day, more nutritious animal diet, and the wound to be dressed with dilute solution of chlorinated soda.

9th. Bowels been affected during night; has some griping pains, but no tenderness on pressure over abdomen; pulse 102; tongue looks well; infusion of hops discontinued; diet restricted to chicken and light vegetable food.

51*

10th. Diarrhœa continues; has had four or five small, light colored and thin passages, containing some mucus, streaked with blood, during the night, accompanied by griping pains. Ordered calomel grs. 3, opium gr. ½, every three hours; gum water to be used freely; diet, boiled milk and rice.

11th. Took three of the pills ordered yesterday; had no passage after taking the first pill, until daybreak this morning, when the diarrhœa returned; had five passages by 8 o'clock; pulse 120; countenance anxious; temples sunken; tongue moist and clean; thirst considerable; skin cool, and tendency to perspiration; griping pains about lower portion of abdomen; no tenderness on pressure. Ordered to continue treatment prescribed yesterday.

12th. Has had four dark thin evacuations by 8 o'clock this morning; countenance looks better; not so much relaxation of skin; pulse 120; ordered sesquinitrate of iron, twenty drops three times a day, and infusion of serpentaria, half a wine-glass full every three hours. 10 o'clock P. M.; passages still frequent; color lighter; *tenderness* over right lumbar, and upper portion of right iliac regions; respiration somewhat laborious.

13th. Evacuations still continue, character same as yesterday; pulse 120 and more feeble; countenance indicative of exhaustion; temples very much sunken; emaciation considerable; wound for the last two days has discharged less freely; edges not so florid. Ordered milk toddy, a table spoonful every two hours; serpentaria and carbonate of iron continued. 2 o'clock P. M. diarrhœa continuing, ordered acetate of lead, grs. ij., opium, gr. ¼ every two hours; arrow root boiled in milk for diet. 10 o'clock P. M.—has had but one evacuation since 2 o'clock; feels more comfortable and less feeble, and has eaten a bowl of arrow root.

14th. Had four evacuations during the night; wound looks more unfavorable, the purulent discharge has almost ceased, and is replaced by a small quantity of dark, thin and offensive fluid, having very much the appearance of the alvine discharges. Treatment of yesterday continued. 10 o'clock P. M.: has had seven or eight passages; pulse more feeble and very rapid; profuse perspiration.

15th. Has had two evacuations during the night, which are rather more consistent, and have a singular *slate color;* countenance rather better; wound discharging as yesterday; the interior of the sac looks dark, and is offensive; the upper and lower portions of the wound have healed, leaving an opening in the middle about two and a half inches in length, communicating with the interior of the sac. Directed that the sac should be syringed out with a solution of chlorinated soda, and the wound dressed with a fermenting poultice; patient to take beef-tea and milk toddy, a table spoonful of each every alternate hour; carbonate of iron as before, and acetate of lead and opium *pro re nata.* 10 o'clock P. M., has had nine small evacuations similar in color to the last, but without pain; has taken 14 grs. acetate of lead, and $1\frac{3}{4}$ grs. of opium since morning.

16th. Diarrhœa continues; wound presents same appearance as yesterday. Ordered calomel grs. iij., camphor grs. ij., opium grs. $\frac{1}{4}$, every three hours; injections of laudanum and starch if diarrhœa is not controlled; stimulus and nutriment as before. 10 o'clock P. M.: has taken four of the pills; has not had the injection; passages not so frequent as yesterday; the last one about the consistence of thick gruel; dark slate color. Pulse more feeble and rapid; skin cool and covered with perspiration; tongue becoming dry, and articulation indistinct. Ordered to double the quantity of stimulus.

17th. Pulse rapid, very feeble and thready; skin more relaxed and cooler; tympanitis has appeared for the first time; all remedies discontinued, except the milk toddy, two table spoonsful of which, to be given every half hour. During the day, the tympanitis increased very rapidly. She gradually sunk and expired about fifteen minutes past 12 o'clock at night.

*Post mortem,* made ten hours after death. Body a good deal emaciated; abdomen very tympanitic. Upon making a section through the linea alba from the sternum down to the fœtal sac, the cavity of the peritoneum was exposed, and, with the slight exceptions presently to be mentioned, was found *free from disease;* there was no effusion either of fluid or of gas in this cavity, while the intestines were greatly distended with the latter.

The viscera occupying the six upper regions of the abdomen were all healthy, and for the most part were found in their usual positions. The viscera occupying the hypogastric and iliac regions, were displaced upwards and backwards by the pressure of the fœtal sac, which lay below and before them, occupying the greater portion of the hypogastric, and a part of the umbilical and the two iliac regions. The small intestines were simply pushed upwards, and were healthy; the cœcum and ascending colon were pushed slightly upwards and backwards; the transverse colon occupied its usual position; the descending colon did not reach its usual inferior limit, but rested upon the left upper portion of the sac, along the whole superior surface of which the sigmoid flexure was extended from left to right, and to which it was firmly adherent. On reaching the right side of the sac, the colon gradually passed behind it, and making its way downwards and to the left, terminated as usual in the rectum. On laying open the lower part of the colon and the rectum, the former was found considerably thickened in consequence of the effusion of lymph, which had caused its adhesion to the sac, the mucous membrane was softened, and, in the rectum, very much congested; a few points of ulceration and some patches of lymph were observed, but there was no communication between the interior of the intestine and the sac.

The sac itself was perfect, presenting no communication with either the abdominal or the pelvic cavity. Its whole internal surface was black and soft, and emitted a most offensive, gangrenous odor. It had contracted very much since the operation, except at its inferior portion, which had probably been distended by the secretions continually gravitating to that point. Its anterior wall was intimately adherent to the anterior parietes of the abdomen; its superior wall supported the sigmoid flexure; its posterior wall was in contact with the rectum.

The uterus and its appendages were found compressed between the lower posterior portion of the bladder and the anterior and inferior wall of the sac. The uterus itself was perfectly normal in form, size and structure, with the exception of three small fibrous tumors found in the substance of its walls; two in the anterior, one in the posterior portion. A small quantity of gelatinous matter was seen protruding from the os tincæ, and extending up the cavity of the neck of the uterus. It presented no indication of previous impregnation. The right ovary was normal, though rather smaller than usual; the right Fallopian tube occupied its usual situation; the fimbriated character of its outer extremity could not be distinguished, owing to effusion around it; its canal was pervious at its uterine extremity for about one half of its length, but was completely impervious along its ovarian extremity. On the left side the Fallopian tube was rendered completely impervious, and the left ovary entirely concealed by the dense effusion of lymph which occupied the whole of the broad ligament of that side.

The mucous membrane of the bladder was congested around the internal orifice of the urethra.

The folds of the peritoneum passing between the uterus and the rectum, were pushed on each side by the fœtal tumor, and presented themselves in the form of large cysts, one on each side, containing eight or ten ounces of yellow serum, mixed with portions of lymph.

Thoracic viscera healthy, with the exception of old plueral adhesions on the right side. The lungs were very much blanched. The heart presented slight deposits of fat on its surface; otherwise normal.

*Remarks.* This adds another to the numerous cases of *abdominal pregnancy* already reported by Diemerbroeck, Esquirol, Moreau, Velpeau and others, and affords another proof, were any now wanting, that impregnation of the ovum and its full development may take place entirely independent of the uterus and the Fallopian tubes. The cause of the escape of the impregnated ovum in this case into the abdominal cavity is probably inexplicable. It is true that the post-mortem examination revealed a condition of things, which, had it existed at the time of impregnation would readily account for it; I mean the occlusion of the Fallopian tubes; but we have every reason to believe that this was the result of inflammation set up *in consequence* of the escape of the ovum into the abdomen, and therefore not concerned in producing that result.

A portion of the history of this case is important. The patient menstruated for the last time about the first of December. Adopting the ovular theory of menstruation, she was in all probability impregnated about the 6th or 7th of December. On the 18th she was seized with symptoms, which the result shows were caused by inflammation of the lower portion of the peritoneum, which, we may fairly conclude, was the result of the attachment of the vivified ovum to the surface of that membrane. The period then from the impregnation to the attachment of the ovum to the inside of the peritoneum was from the 7th to the 18th of December, an interval of 11 days. I am not aware that any case hitherto reported establishes this fact. This case also contradicts the statement of Cazeau, (*Cazeau's Midwifery*, 2d part, p. 144,) that in cases of *primary abdominal pregnancy, no adventitious sac* is thrown around the ovum, who explains this supposed fact by stating in the language of M. Deseimeris, " qu' un corpuscle aussi peu volumineux, aussi simple, aussi fragile, ne peut provoquer qu' une excitation très légère sur lequel il s'arrête, et que le champ de cette excitation ne peut dépasser les limites du contact du petit corps étranger." That the advent of " this little foreign body" into the cavity of the abdomen was sufficient to cause very violent inflammation was proved by the attack which the patient had on the 18th of December, and that this resulted in the formation of a distinct and complete sac was proved by the result of the operation and of the post mortem examination.

Another interesting point in the history of this case is the unusual length of time that the fœtus must have lived. Velpeau (Dictionnaire de Médecine, vol. 14, Art. Grossesse Extra-Uterine,) says, "that extra-uterine pregnancy, usually terminates before the fifth month by the death of the fœtus or by the rupture of the cyst;" and again, "It is rare that the fœtus continues to live beyond the 3d or 4th month." In this case the development was fully as great as at ordinary term, and the weight of the fœtus was nearly two pounds greater than the average weight of a full grown fœtus. May not indeed the fœtus have continued to be nourished and to grow, subsequently to the termination of the ninth month? I can conceive of no reason why it should not, unless it be that the compression to which it was subjected by the contraction which took place at the close of the ninth month was sufficient to cut off its circulation, and thus to produce its death; and this we can scarcely suppose, as these contractions were exerted upon a sac filled with fluid, and were moreover so feeble as not to produce a separation of any portion of the placenta. The cause of death in these cases previous to the rupture of the sac is a most interesting subject, and one upon which the profession has but little information. In opposition to the views of Velpeau, the statistics given by Dr. Campbell in his essay on this subject, would show that it is not uncommon for the fœtus to live during the full term of utero-gestation. "In ninety-eight cases," he says, "in which we can decide, or nearly so, on the period of pregnancy, the fœtus in

*seventy-nine* patients died at the close of nine months, or soon thereafter."

The patient stated that she distinctly felt *what she conceived to be the movements of the child*, up to within a short time before the operation, not longer, she was sure, than six weeks. Now from what has been already stated with regard to the condition of the fœtus and the placenta, it is highly improbable that the child was not dead some months before that period. If so, then these movements must have been produced otherwise than by the child, chiefly no doubt by the abdominal parietes; partly, it is possible by the sac itself. This fact sustains, as far it goes, the ingenious views, lately promulgated on this subject, by Dr. Tyler Smith, and goes far to discredit, the value of these movements as diagnostic signs of the life of the child.

I conclude this paper with one other remark. Under the circumstances, ought the operation to have been performed, or would it have been better to have left the case entirely to the management of nature? When we look at the result of the case, and find ourselves forced to the melancholy conclusion that, through our agency, the life of this poor woman has been certainly shortened, we naturally shrink from the immense responsibility which the discharge of our professional duty, as we have conceived it, has devolved upon us. In deciding, however, upon the propriety of a serious operation, the only questions which the surgeon has to consider are, whether, 1st, the operation is necessary to the well being of the patient; and 2d, whether the patient is willing to encounter the attendant risk for the chance of ultimate recovery. In this case the patient was perfectly willing to have the operation done, after being fully apprised of its danger. That the operation was necessary for the well-being of the patient, I infer from the history of a vast majority of those cases which have been suffered to run their course without interference—ulceration, discharge of a portion of the contents of the sac, and death from irritative fever, briefly describing them. That such would have been the result in this case, and that within a short period, I infer from the decomposition of the fœtus and from the adhesions between the colon and the sac which had taken place. The only hope therefore of permanently relieving the patient was by the operation, and the period selected for it was the most favorable which had occurred since her impregnation. All danger from hemorrhage was avoided by the impervious condition of the placental blood-vessels--the sac was still perfect, and could therefore be incised without any danger of injuring the proper cavity of the peritoneum, and the general health of the patient, recovered from the previous attacks of peritoneal inflammation, had not yet begun to suffer from the effects of the decomposition of the fœtus. Under the circumstances, I conceive that the operation was not only justifiable, but was demanded as promising a very fair chance of ultimate recovery, to one who, without it, was doomed to a certain and miserable death. By reference to the history of the case, it will be seen that up to the 9th of June, *sixteen days* after the operation, the patient continued to do well. At this period, a marked change took place in the weather, producing a decided tendency to affections of the alimentary canal throughout our city. Is it not fair to conclude that this cause, acting upon an individual predisposed to such an affection by the immediate contiguity of a large suppurating sac, was mainly instrumental in producing the fatal result? In this connection Velpeau (*loc. cit.*) says—"It is true that, practiced in desperate cases, as it" (gastrotomy) "has hitherto been, it has not always prevented death. I think, however, with Desormeaux, that if had recourse to early, when the formidable assemblage of inflammatory symptoms, has not yet been developed, before peritonitis itself constitutes a fatal disease, it will save a great number of women." Again, "with the operation, death is but too probable; without it, it is nearly certain." And Colombat (Diseases of Females, p. 583) says, "we believe that the operation ought to be performed even after the rupture of the cyst, and that in general we ought not to wait until the symptoms of peritonitis shall have developed themselves, because in that case we are almost sure to see the mother and child perish, when, by operating earlier, we might, *perhaps, have saved both.*"

NOTE.—According to the statistics given by Dr. Campbell in his essay above referred to, the operation of extraction in these cases has been remarkably successful. He tells us, "that of thirty cases in which gastrotomy was performed, *twenty-eight* patients recovered. Such success, greater than that which attends ordinary amputations of the extremities, would afford a strong indication for the operation."

